# Statistical Inferences Using Effect Sizes in Human Endothelial Function Research

**DOI:** 10.1007/s44200-021-00006-6

**Published:** 2021-10-27

**Authors:** Joshua M. Cherubini, Maureen J. MacDonald

**Affiliations:** Department of Kinesiology, Vascular Dynamics Lab, McMaster University, Ivor Wynne Centre, Room E210, 1280 Main Street West, Hamilton, ON L8S 4K1 Canada

**Keywords:** Endothelial function, Statistics, Effect sizes, Statistical power

## Abstract

**Introduction:**

Magnitudes of change in endothelial function research can be articulated using effect size statistics. Effect sizes are commonly used in reference to Cohen’s seminal guidelines of small (*d* = 0.2), medium (*d* = 0.5), and large (*d* = 0.8). Quantitative analyses of effect size distributions across various research disciplines have revealed values differing from Cohen’s original recommendations. Here we examine effect size distributions in human endothelial function research, and the magnitude of small, medium, and large effects for macro and microvascular endothelial function.

**Methods:**

Effect sizes reported as standardized mean differences were extracted from meta research available for endothelial function. A frequency distribution was constructed to sort effect sizes. The 25th, 50th, and 75th percentiles were used to derive small, medium, and large effects. Group sample sizes and publication year from primary studies were also extracted to observe any potential trends, related to these factors, in effect size reporting in endothelial function research.

**Results:**

Seven hundred fifty-two effect sizes were extracted from eligible meta-analyses. We determined small (*d* = 0.28), medium (*d* = 0.69), and large (*d* = 1.21) effects for endothelial function that corresponded to the 25th, 50th, and 75th percentile of the data distribution.

**Conclusion:**

Our data indicate that direct application of Cohen’s guidelines would underestimate the magnitude of effects in human endothelial function research. This investigation facilitates future a priori power analyses, provides a practical guiding benchmark for the contextualization of an effect when no other information is available, and further encourages the reporting of effect sizes in endothelial function research.

## Introduction

Changes in arterial vasomotion and calibre are, in large part, mediated by a functioning vascular endothelium that senses mechanical, tensegral, and molecular stimuli in the vessel lumen [1]. Measures of endothelial function are therefore commonly derived from data that quantify changes in vasomotor capacity and blood flow [2, 3]. Such data can be treated using traditional frequency-based statistics which are common practice in the biomedical sciences [4]. Frequency-based statistical methods facilitate binary interpretations of outcomes, and enable scientists to reject, or fail to reject, the null hypothesis [5–7]. While these analyses have both practical and theoretical value [8], researchers are often interested in assessing and reporting on the *magnitude* of effects; particularly as they pertain to outcomes in the biomedical sciences, including endothelial function.

**Fig. 1:**
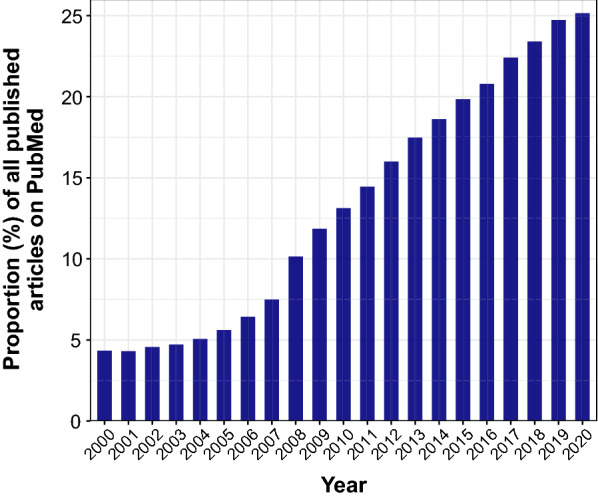
Increased interest in effect sizes in scientific research over the last two decades as evidenced by articles containing the keyword ‘effect size’ in PubMed. Figure created using the ‘EuropePMC’ package [41]

The use of effect sizes in biomedical literature has increased over the last two decades (Fig. 1). Effect sizes, such as standardized mean differences (SMD), facilitate a priori power analyses, articulate the magnitude of effects, and encourage the progression of science regardless of the units of the original outcome measures [6, 9]—an especially relevant feature given the range of methods [2] and calculations [3] that are used for the representation of endothelial function [6, 10]. Despite the burgeoning popularity of effect size assessment and reporting, an efficient and standardized interpretation of effect sizes are limited by a paucity of guiding recommendations [9]. Durlak [10] eloquently states that the best way to interpret effect sizes is to use context-specific and practical interpretations that are guided by the uniqueness of an experimental architecture. Failing this standard, it is also useful to contextualize an effect using indices derived from the broader field of literature [9, 11, 12]. Seminal benchmarks for small (*d* = 0.2), medium (*d* = 0.5), and large (*d* = 0.8) effects were established by Cohen [11], who reasoned that these values serve as a last-resort interpretation for effect sizes in the absence of any other useful information [10, 12]. These benchmarks were developed for use in the behavioural sciences and described according to the extent to which the effect magnitudes were visually perceptible [11–13]. Empirical analysis of effect size distributions in various sub-disciplines of psychology [14], as well as in the biomedical sciences [12, 15–17], have shown distributions that differ from Cohen’s recommendations.

We, therefore, examined effect size distributions in endothelial function to facilitate future a priori power analyses, provide a practical guiding benchmark for the contextualization of an effect when no other information is available, and further encourage the reporting of effect sizes in human endothelial function research.

## Materials and Methods

We adhered to methods that have been reported in prior research works [12, 14–17]. All analysis was conducted using the R statistical environment [18], and the code and original data to reproduce this work is made publicly available at the following online repository: https://doi.org/10.5683/SP2/BEVNRG.

### Data Extraction and Preparation

Meta-analyses were identified through a PubMed search conducted in April 2021 using the following search terms: [“endothelial function” (Title/Abstract)] AND [meta-analy*(Title/Abstract)]. Meta-analyses were screened for eligibility, and the included meta-analyses are accessible as supplementary material. Effect sizes, and their corresponding lower and upper limits, were extracted from any meta-analysis that reported a standardized mean difference effect size (SMD; *Cohen’s d* or *Hedges’ g*) for endothelial function assessed via changes in vasomotion or changes in blood flow. Meta-analyses that included outcomes related to pulse-wave velocity or augmentation index were excluded as these outcomes, though correlated with measures of endothelial function, also represent measures of arterial stiffness and structure and therefore beyond the scope of this work [19]. We reported the absolute value of the effect sizes as the objective of our investigation was to query the magnitude, and not direction, of the reported effects for the effect size distribution analyses [12, 15].

Group sample sizes and the region of vascular assessment (microvascular or macrovascular) were extracted from descriptive tables or figures in the meta-analysis. In the event that this information was missing or ambiguous, the details were either retrieved from the primary article or coded as unavailable. We also recorded the category of the studied biological process that corresponded to the effect size extracted from the meta-analyses to appreciate the range of investigation topics included in the data. The biological process categories were determined according to the title and topic of the meta-analysis.

A *Hedges’ g* correction was applied to provide a conservative estimation of *Cohen’s d* values; particularly for those based upon small sample sizes or predicated upon biased sample size estimates [6]. *Cohen’s d* values were converted to *Hedges’ g* using a formula derived from Lakens [9], where *n*_*1*_ and *n*_*2*_ denote group sample sizes:

$$Hedges^{\prime}g = Cohen^{\prime}s ~d \times \left( {1 - \frac{3}{{4\left( {n_{1} + n_{2} } \right) - 9}}} \right)$$

We also solved for *Cohen’s d* from the above equation to enable us to convert effect sizes that were originally reported as *Hedges’ g* in meta-analyses to *Cohen’s d*, such that all effect sizes could be expressed as either a *Cohen’s d* or *Hedges’ g* effect statistic. If a *Hedges’ g* effect size was reported with no indication of sample size, then the statistic was kept as *Hedges’ g* in our analysis [14].

Duplicate effect sizes were coded as such and subsequently removed from analysis. Effect sizes larger than eight were deemed to be unrealistically large and classified as outliers, and therefore removed from our analysis to mitigate the influence of outlying data on effect size distributions. We chose to classify effect sizes larger than eight as outliers because this value corresponded to the largest effect size reported among 6,447 effect sizes from social psychology [14].

### Statistical Analyses

Cohen’s original work defined that small and large effect sizes are equidistant from the medium effect size [12, 20]. Thus, we rank-ordered and plotted effect sizes from smallest to largest using a frequency distribution and found the effect sizes at the 25th, 50th, and 75th percentiles. We then subdivided the data according to the assessment of vessel type (macrovascular or microvascular), and according to the modality used to measure endothelial function [ultrasonography, plethysmography (peripheral arterial tonometry was included in this category due to its classification as a plethysmographic method [21, 22]), laser doppler, or other measures such as capillaroscopy and near-infrared spectroscopy] and repeated the aforementioned analysis. The ‘psych’ package [23] was used to describe the skew and kurtosis of each distribution.

We examined the bivariate relationships between effect size magnitude and year, and effect size magnitude and the logarithm of sample size. We plotted the reported effect sizes as a function of the year in which they were published to visualize any changes in the magnitude of effect sizes over time, as measurement methods and guidelines for endothelial function change over time [24, 25]. A locally estimated scatterplot smoothing curve with a smoothing parameter span set to ƒ = 0.75 was employed to visualize the relationship between effect size magnitude and year of publication. Locally estimated scatterplot smoothing was used to detect any fluctuation in effect size magnitude associated with the publication of guidelines for the assessment of endothelial function using flow-mediated dilation (in 2011, see [24]; and in 2019, see [25]). Some meta-analyses reported multiple effect sizes from the same study and thereby include correlated observations that violate assumptions of independence. To mitigate the undue influence of correlated data, we computed regression coefficients (*β*) and adjusted standard errors (SE_*adj*_) using vce(cluster) syntax on STATA (version 17; StataCorp, College Station, TX, USA) as well as a clustered bootstrap 95% confidence intervals (95% CI) based on 10,000 samples using the ‘jtools’ [26] and ‘ClusterBootstrap’ [27] packages available in the R environment.

A series of a priori power calculations for independent and paired samples *t* tests, powered to detect the small, medium, and large effect sizes for endothelial function research, were computed using the ‘pwr’ package [28]. A visualization of the sample sizes required to reliably detect a given effect size, for a range of statistical power, for independent samples and paired *t* tests was constructed. Each power calculation was determined assuming two-tailed analyses and *α* set to 0.05.

The ‘metameta’ package [29] was used to visualize the median power of all meta-analyses that are included in our work. The evidential value of each meta-analysis was displayed across a range of possible true effect sizes from *δ* = 0.1 to *δ* = 1.0, as well as for the observed summary effect size reported in the meta-analysis.

## Results

**Fig. 2:**
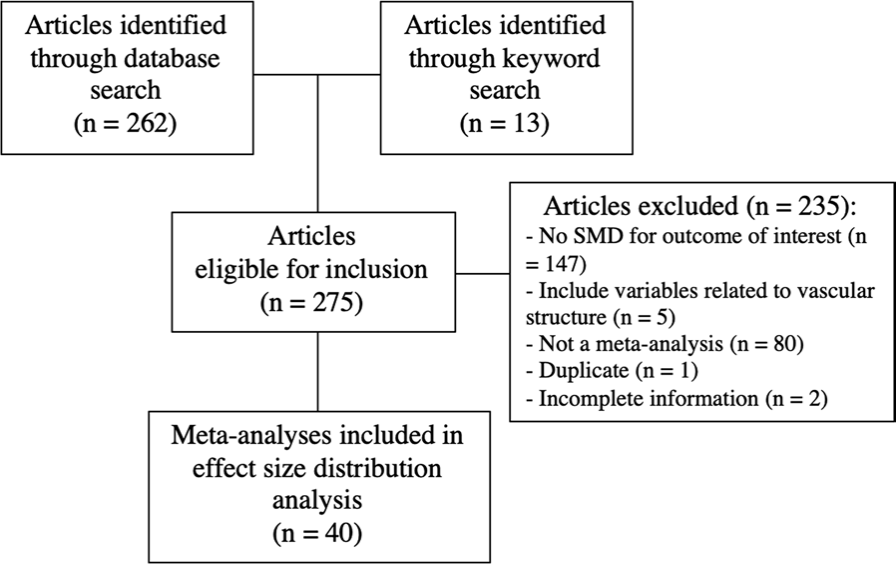
Flowchart depicting meta-analysis inclusion based on a PubMed search query entered April 2021. *SMD* standardized mean difference

The PubMed search query produced a total of 262 scientific articles. An additional thirteen articles were identified using keyword searching, thereby resulting in 275 articles that were assessed for eligibility (Fig. 2). After screening, 40 meta-analyses containing 760 effect sizes satisfied our eligibility criteria and were included in this analysis. After duplicate effect sizes (*n* = 6), and outlying effect sizes larger than eight (*n *= 2) were removed, 752 unique effect sizes were extracted and included in the analysis.

### Effect Size Distributions

**Fig. 3:**
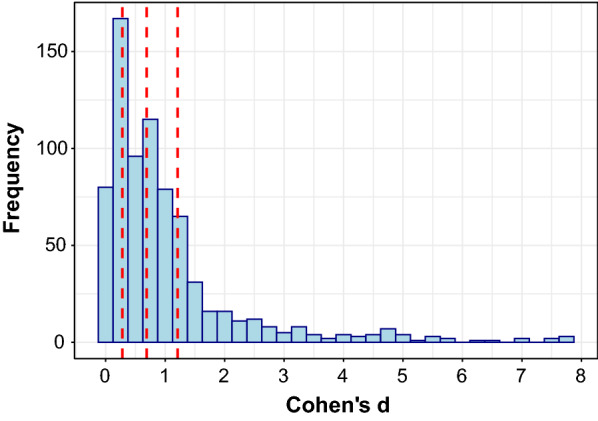
Histogram depicting the effect size distribution for endothelial function research. Vertical red lines at the 25th, 50th, and 75th percentiles correspond to small (*d* = 0.28), medium (*d* = 0.69), and large (*d* = 1.21) *Cohen’s d* effect sizes

**Fig. 4:**
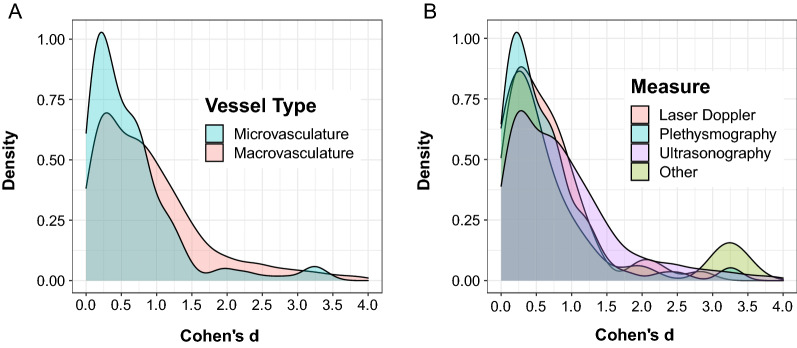
Panel A depicts a density plot of the effect size distributions for macrovascular (*n* = 544; skewness = 2.61; kurtosis = 8.27) and microvascular (*n* = 205; skewness = 2.99; kurtosis = 9.85) endothelial function. Panel B depicts a density plot of the effect size distributions for different measures used to assess endothelial function such as ultrasonography (*n *= 559, skewness = 2.64, kurtosis = 8.53), plethysmography (*n *= 98, skewness = 3.23, kurtosis = 12.08), laser doppler (*n *= 60, skewness = 2.85, kurtosis = 8.39), and other measures (*n *= 29, skewness = 1.67, kurtosis = 1.74). Cohen’s *d* values greater than 4.0 were not shown for visualization purposes

We first analyzed the overall effect size distribution for endothelial function research (Fig. 3). We found that the 25th, 50th, and 75th quantiles corresponded to Cohen’s d effect sizes of *d* = 0.28 (small), *d* = 0.69 (medium), and *d* = 1.21 (large), respectively. Next, we separated the data according to the vessel bed that was analyzed, being either macrovasculature or microvasculature, and repeated the aforementioned distribution analysis. We found different effect size distributions from the arterial region subgroup analysis for macrovascular (*n* = 544; skewness = 2.61; kurtosis = 8.27) and microvascular (*n* = 205; skewness = 2.99; kurtosis = 9.85) endothelial function. The small (macrovascular *d* = 0.34; microvascular *d* = 0.20), medium (macrovascular *d* = 0.76; microvascular *d* = 0.49), and large (macrovascular *d* = 1.31; microvascular *d* = 0.90) effect sizes for the macrovasculature were consistently larger than that of the microvasculature (Fig. 4A). We also grouped data according to the method used to estimate endothelial function, including plethysmographic techniques (n = 98, skew = 3.23, kurtosis = 12.08), laser doppler (*n *= 60, skew = 2.85, kurtosis = 8.39), ultrasonography (*n *= 559, skew = 2.64, kurtosis = 8.53), and other methods (*n *= 29, skew = 1.67, kurtosis = 1.74). The effect size distribution for plethysmography (small = 0.20, medium = 0.48, large = 0.84) revealed magnitudes similar to Cohen’s guidelines whereas all other imaging modalities produced small, medium, and large magnitudes that were comparatively larger (Fig. 4B). We identified four categories that represent the range of biological processes that were studied in the included effect sizes. A distribution depicting these four categories, including exercise (*n *= 150), pharmacological supplementation and dietary interventions (*n *= 280) pathophysiological processes (*n *= 273), and other processes (such as menstruation and behavioural interventions; *n *= 49), is included as supplementary data.

### Correlates of Effect Size

**Fig. 5:**
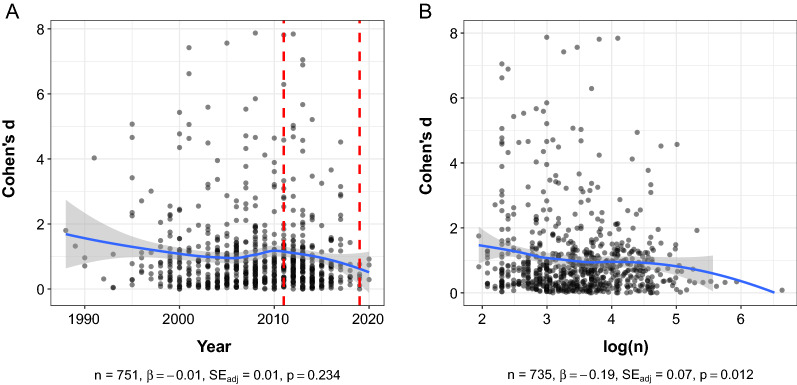
Correlates of effect sizes in endothelial function research. Panel A shows no relationship between *Cohen’s d* and the year of primary study publication. The vertical red lines indicate the years in which the guidelines for the assessment of macrovascular endothelial function were published. Panel B depicts a weak negative relationship between *Cohen’s d* and the logarithm of sample size

We sought to investigate differences in the magnitude of reported effect sizes for endothelial function across the individual study publication year and sample size. While locally estimated scatterplot smoothing showed a slight upward inflection in effect size magnitude around 2009 and a downward inflection in effect size magnitude around 2011 (Fig. 5A), the reported effect sizes generally remained consistent over time [*β* =  − 0.01, *p* = 0.234, SE_*adj*_ = 0.01, 95% CI (− 0.03, 0.01)]. A weak negative relationship was observed between the magnitude of reported effect size and logarithm of sample size [*β* =  − 0.19, *p* = 0.012, SE_*adj*_ = 0.07, 95% CI (− 0.33, − 0.04)]. Clustered bootstrapping procedures did not change any interpretations of the aforementioned bivariate relationships; no association between effect size and year [bootstrapped 95% CI (− 0.03, 0.01)], and a weak negative relationship between effect size and the logarithm of sample size [bootstrapped 95% CI (− 0.35, − 0.06)] persisted.

### A priori Power Analyses

**Fig. 6:**
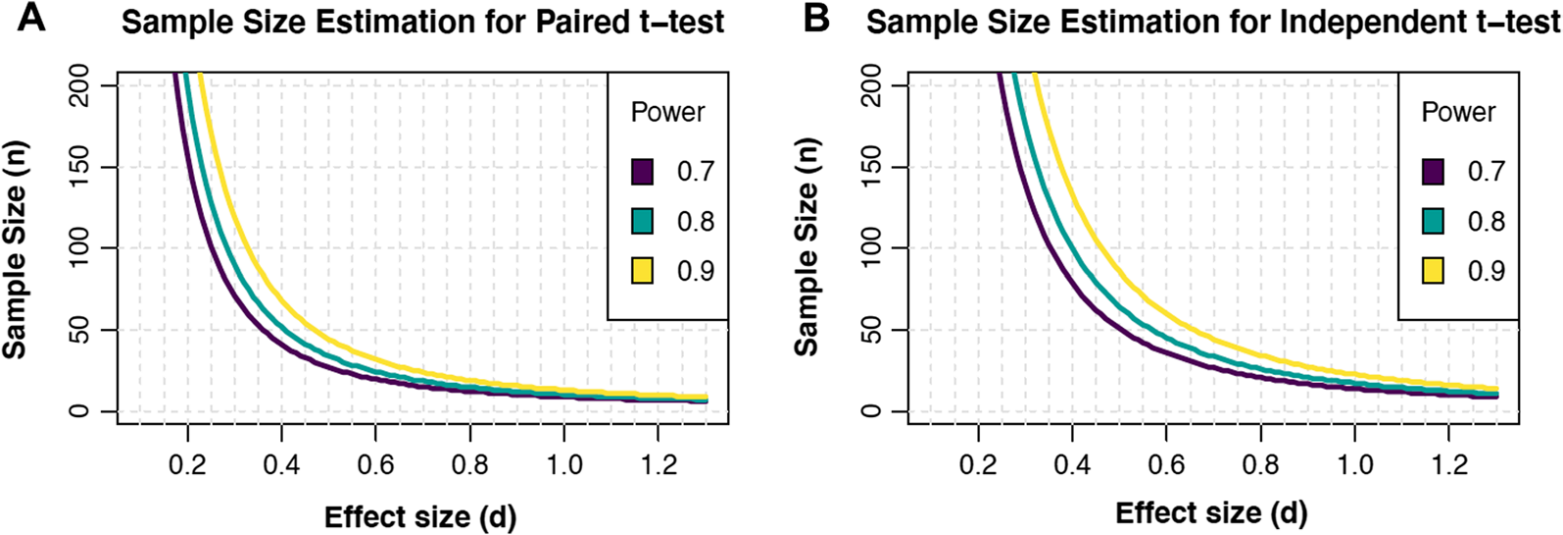
Relationship between sample size (*n* = number of pairs) and effect size for a paired samples *t* test (Panel A), and sample size (*n* = number in each group) and effect size for independent samples *t* test (Panel B), across three different levels of statistical power. Fewer participants per group are required for a given effect size using the paired samples *t* test. Figures assume a two-tailed analysis, with *α* = 0.05. Adapted from Yu and Yagle [42]

The median sample sizes for treatment and control groups in endothelial function research were 21 and 20, respectively. Assuming a type 1 error rate of 5% and a two-tailed analysis, these sample sizes allow the reliable detection (or rejection) of large effects only (*d* = 1.21, power = 0.965). These median sample sizes are, however, inadequately powered to detect the presence of small (*d* = 0.28, power = 0.141) and medium effects (*d* = 0.69, power = 0.577) in endothelial function research. We present a priori power analysis calculations for independent and paired samples *t* tests based on our derived effect size distributions in Table 1. Figure 6 provides a visualization of the relationship between sample size and effect sizes that can be reliably detected using 80% statistical power.

**Table 1:** a priori sample size calculations to detect small, medium, and large effects for endothelial function research

Effect size	Paired samples *t* test (*n *= number of pairs)	Independent samples *t* test (*n *= per group)
Small (*d* = 0.28)	102	202
Medium (*d* = 0.69)	19	34
Large (*d* = 1.21)	8	12

Tests were calculated using 80% power, and assuming a two-tailed test and *α *= 0.05. Researchers may use alternative levels of statistical power (Fig. 6), however, 80% typically represents the minimally acceptable level of statistical power (Quintana [12])

### Statistical Power of Meta-Analyses

**Fig. 7:**
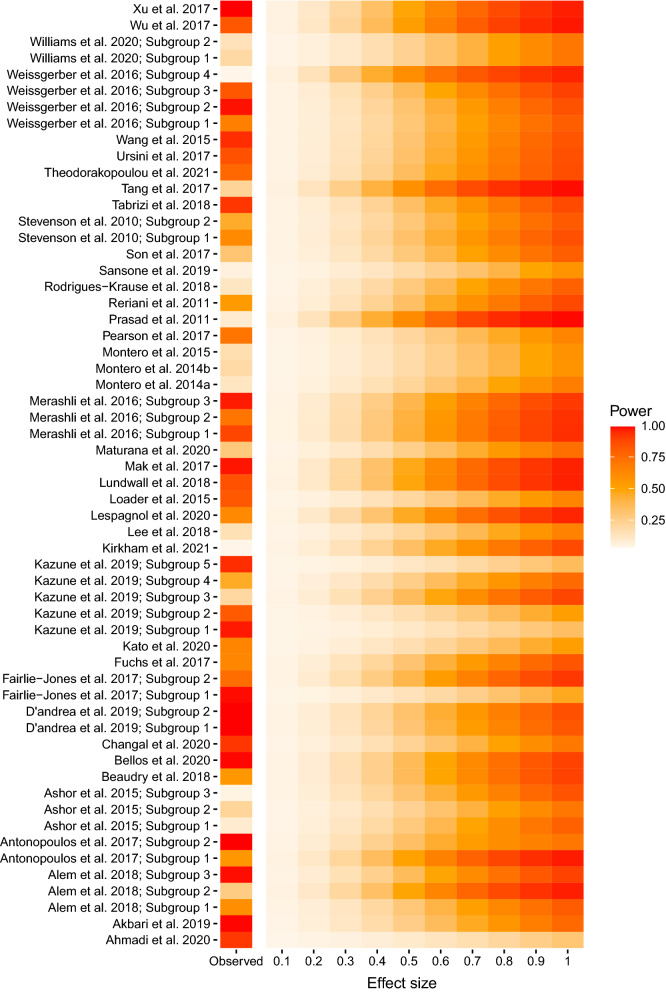
The statistical power of the observed summary effect size in each of the included meta-analyses, and the median statistical power for included meta-analyses displayed across a range of plausible true effect sizes (from *δ *= 0.1 to *δ* = 1)

The median reported summary effect size across all of the included meta-analyses was 0.14 (mean =  − 0.08, min =  − 6.26, max = 2.41). We found that the median statistical power to detect the reported summary effect size was approximately 66.6% (mean = 60.7%, min = 5.0%, max > 99.9%). The median power to detect a range of plausible true effect sizes, from *δ* = 0.1 to *δ* = 1.0, across the included meta-analyses is displayed in Fig. 7 [30]. Our data shows that most meta-analyses in both microvascular and macrovascular endothelial function research can reliably detect medium-to-large summary effect sizes, as the median power for an assumed true effect of *δ* = 0.9 was approximately 76.8% (mean = 72.6%, min = 26.5%, max = 98.4%). The corresponding power to detect the entire range of plausible true effect sizes from *δ* = 0.1 to *δ* = 1.0 is made available in the supplementary data.

## Discussion

We report a novel effect size distribution for endothelial function research and identify small (*d* = 0.28), medium (*d* = 0.69), and large (*d* = 1.21) effects that correspond to the 25th, 50th, and 75th percentiles of effect sizes reported in endothelial function research. These values may serve as a benchmark to interpret effect sizes specific to endothelial function in the absence of any other context or information. Our analyses also indicate that future studies investigating endothelial function may benefit from higher-powered statistical designs so that small and medium effects can be reliably detected.

Cohen originally proposed *d* = 0.2, *d* = 0.5, and *d* = 0.8 to constitute small, medium, and large effects. These benchmarks were originally intended for the psychological sciences and described in terms of the extent to which they are visually perceptible [11, 12]. Empirically derived effect size distributions within the biomedical sciences differ from Cohen’s proposed guidelines. For instance, effect size distributions derived from rehabilitation literature, gerontology, and heart rate variability case–control studies show both overestimations and underestimations of what Cohen classified as small, medium, and large effects [12, 15, 17]. These observations, together with our derived benchmarks for endothelial function research, support the previously reported notions that Cohen’s guidelines may not be generalizable to other biomedical fields. Therefore, researchers interested in contextualizing their results within the broader field of endothelial function research may, instead, be guided by the effect size distribution and indices that we provide.

Different distributions in effect magnitude were observed between macrovascular and microvascular endothelial function (Fig. 4A). The distribution for macrovascular endothelial function had a smaller skew and kurtosis than the distribution for microvascular endothelial function, indicating a higher density of small effect sizes in the microvasculature compared to the macrovasculature. We speculate that this disparity is attributable to the heterogeneity that exists in methods used to assess endothelial function. Indeed, differences in endothelial function based on the method of assessment have been previously described [31]. Molecular differences in signal transduction mechanisms within the micro- and macrovascular endothelium have been extensively reported. For instance, the dilatory influence of endothelial-derived hyperpolarizing factors have been shown to be more prominent within the microvasculature [2, 32]; whereas nitric oxide is a large contributor to vasodilation in the macrovasculature [33]. It may be that heterogeneity in assessment methods and mechanisms contribute to the observed differences in the reported effect size distributions, however, a thorough description of these methods and mechanisms is beyond the scope of this work. We direct interested readers to further research that discusses the methods used to assess microvascular and macrovascular endothelial function, and how these methods and mechanisms may associate with differing vascular responses [2, 34, 35].

We observed that the magnitude of reported effect sizes in human endothelial function research remained constant over a range of publication years, despite the introduction of different guidelines for methods of assessing endothelial function. Given that the most recent guidelines for the assessment of endothelial function were released in 2019, more time may be needed for any impact of those guidelines on effect magnitude to manifest. We also found a weak negative relationship between sample size and effect size. This finding is consistent with the relationships between sample size and effect magnitude as identified in previous reports [13, 14]. That the negative relationship between sample size and effect magnitude persists even amongst pre-registered studies [13] suggests that publication bias, or potential ‘file drawer’ effects, may not be the principal factor underlying this finding [36]. Schäfer and Schwarz [13] speculate that the observed relationship may also be attributable to the fact that larger sample sizes are required to detect smaller effects, and thus the contrary, that smaller samples are generally sufficient to detect larger effect sizes.

Much existing endothelial function research may be underpowered to detect a plausible range of true effect sizes; particularly those of small and medium magnitude. The median sample size for treatment and control groups in endothelial function research is similar to those reported in heart rate variability and biomedical gerontology [12, 15], and sufficient only to detect the large effects for endothelial function that we report herein. Furthermore, the median power of meta-analyses included in this investigation indicates that most meta-analyses in endothelial function research can only reliably detect medium-to-large-sized effects. Future research in endothelial function should consider the use of techniques that increase statistical power. Augmented and effective definitions of statistical power can be achieved, for instance, by heeding suggestions to increase group sample sizes when appropriate, or by using sequential testing methods, described in further detail elsewhere [37, 38]. We also include Fig. 6 which shows the sample sizes required to detect a given *Cohen’s d* effect size across a range of type 2 error rates (*β* = 0.7, 0.8, and 0.9; *α *= 0.05), for paired and independent samples *t* tests. Improving statistical power will bolster the quality of the interpretation of significant and non-significant results and increase the likelihood of replicable results in endothelial function research.

Several limitations in this investigation warrant acknowledgement. First, effect size distributions provide an efficient and practical guide to interpret effect magnitudes and for a priori power analysis. However, this work should not be interpreted as an endorsement to exclusively interpret endothelial function research in the context of the reported effect size benchmarks. Priority should always be given to experiment-specific interpretations of effect magnitude since each experimental question and design is unique [10]. Second, pre-registered publications have been shown to produce smaller effect size estimates, and thus, a lack of pre-registered studies in endothelial function research may distort the presented distributions [36]. Therefore, it would be interesting to repeat our analysis as pre-registration becomes more popular in endothelial function research and to note differences in effect size distributions between pre-registered studies and studies without pre-registration [13]. Lastly, we included effect sizes reported as standardized mean differences from meta-analyses (*Cohen’s d* and *Hedges’ g*), and excluded other statistics such as risk ratios and correlation coefficients. We also followed previous analyses [14–16] and included effect sizes from different study designs in our investigation. As first noted by Quintana [12], the inclusion of different study designs may introduce different biases into the results and effect size distributions; particularly considering the calculation of effect sizes from repeated measures data where the correlation between outcomes is unknown [39]. However, it is also important to acknowledge the robustness and utility inherent in effect sizes derived from repeated measures, and that excluding effect sizes on the basis of study design may also eliminate potentially valuable information [40]. Nonetheless, and in accordance with precedent established by Quintana [12] and Kinney, Eakman, and Graham [17], we believe a large number of effect sizes and study designs included herein to be sufficiently representative of the distribution of standardized mean differences reported in human endothelial function research.

## Conclusion

Our analysis serves as a benchmark to contextualize the magnitude of effects within endothelial function literature, in addition to experiment-specific interpretations of the effect size relevance. This work will facilitate a priori power analyses and sample size calculations when no other information is available to determine a small, medium, or large effect. This report contributes to a general appreciation for the effect size distributions in endothelial function research and may consequently encourage the use of effect sizes in the endothelial function literature.

## Data Availability

The data and analysis script used for this investigation are provided in an online repository: https://doi.org/10.5683/SP2/BEVNRG
